# A case of *Exophiala oligosperma* successfully treated with voriconazole^☆^

**DOI:** 10.1016/j.mmcr.2013.08.003

**Published:** 2013-09-08

**Authors:** Bassam H. Rimawi, Ramzy H. Rimawi, Meena Mirdamadi, Lisa L. Steed, Richard Marchell, Deanna A. Sutton, Elizabeth H. Thompson, Nathan P. Wiederhold, Jonathan R. Lindner, M. Sean Boger

**Affiliations:** a Medical University of South Carolina, Charleston, SC 29414, USA; b Brody School of Medicine – East Carolina University, Greenville, NC 27834, USA; c University of Texas Health Science Center at San Antonio, San Antonio, TX 78229, USA

**Keywords:** *Exophiala oligosperma*, Fungus, Voriconazole, Phaeohyphomycosis, Mycetoma

## Abstract

*Exophiala oligosperma* is an uncommon pathogen associated with human infections, predominantly in immunocompromised hosts. Case reports of clinical infections related to *E. oligosperma* have been limited to 6 prior publications, all of which have shown limited susceptibility to conventional antifungal therapies, including amphotericin *B*, itraconazole, and fluconazole. We describe the first case of an *E. oligosperma* induced soft-tissue infection successfully treated with a 3-month course of voriconazole without persisting lesions.

## 1 Introduction

*Exophiala* is a genus of anamorphic fungi in the family *Herpotrichiellaceae* [1]. The primary species causing human disease is *Exophiala dermatitidis*. Isolates exhibit marked neurotropism and are rare agents of severe, life-threatening cerebral phaeohyphomycosis, primarily in Southeast Asia. The species is more commonly recovered from respiratory, cutaneous, and subcutaneous sites, and occasionally from other deep-seated infections. However, species recently noted to be recovered from clinical samples, such as *Exophiala oligosperma* and *Exophiala xenobiotica*, have also been reported as agents of systemic phaeohyphomycosis [2]. Mycetoma and phaeohyphomycosis are the most common infections associated with this opportunistic pathogen. Although the majority of these are non-life-threatening cutaneous infections, including subcutaneous cystic lesions and soft-tissue abscesses, fatal systemic infections have also been described. Case reports of clinical infections related to this organism have been limited to 6 prior publications. We describe the first case of an *E. oligosperma* induced soft-tissue infection successfully treated with a 3-month course of voriconazole without persisting lesions requiring concomitant surgical intervention.

## 2 Case

A 50-year-old Caucasian female presented in March of 2012 with progressively enlarging, painless, “rubbery-like” cutaneous lesions on both of her legs over the past year. Although she noted occasional thorn pricks from different plants while gardening, she denied any prior major trauma to her legs. Occasionally, the lesions spontaneously drained black, purulent fluid.

**Fig. 1:**
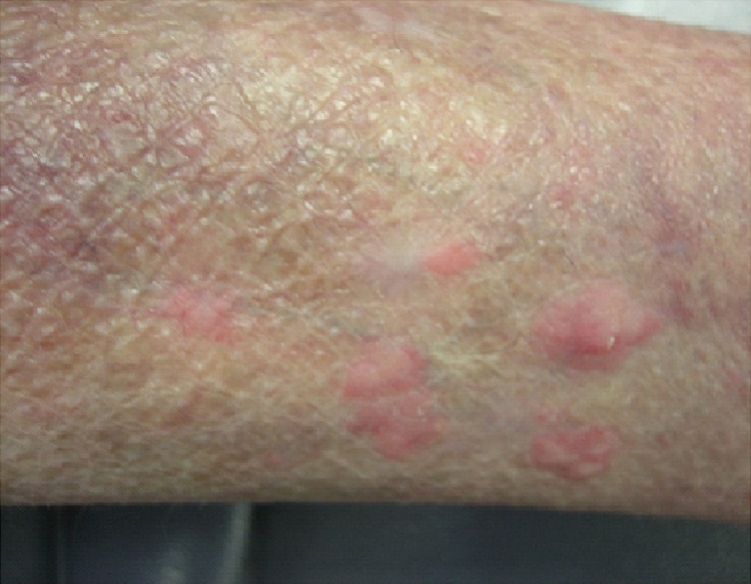
Before voriconazole therapy – showing nodular and diffuse dermatitis.

She has a 10-year history of systemic lupus erythematosis on chronic immunosuppressive therapy with mycophenolate mofetil 500 mg daily and plaquenil 200 mg daily. She also has a history of severe asthma and had been taking oral methylprednisolone 80 mg daily for years. Her vital signs were normal. She was afebrile and hemodynamically stable. Her physical exam was unremarkable except for the skin examination, which revealed multiple, tender, raised, reddish-pink subcutaneous nodules with some measuring 0.5–1 cm in diameter, while others were up to 3 cm in diameter, all grouped in the affected zone (Fig. 1). There was no lymphadenopathy.

**Fig. 2:**
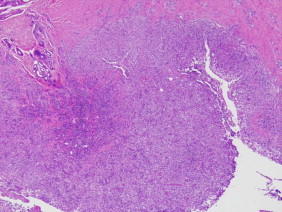
H&E stain demonstrating suppurative granulomatous inflammation in the deep dermis and hypodermis composed of lymphocytes, epithelioid macrophages, multinucleated giant cells and aggregations of neutrophils in the center of the granulomas.

**Fig. 3:**
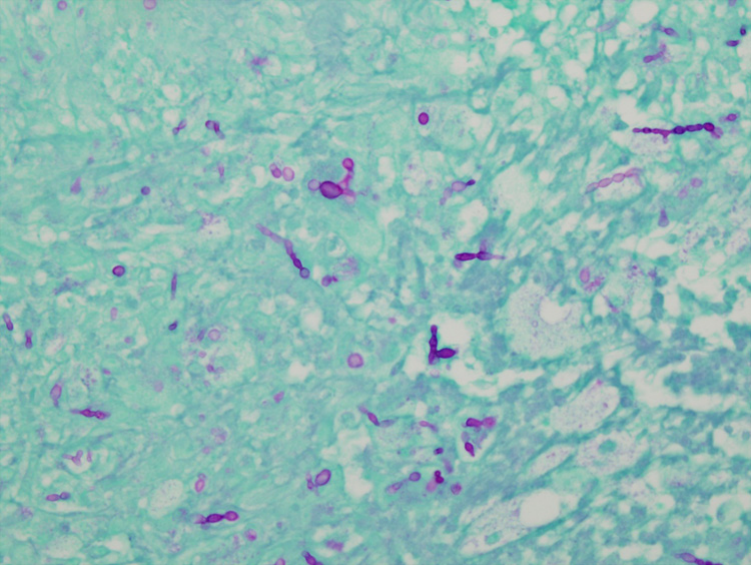
Periodic acid-Schiff (PAS) stain of a punch biopsy from the proximal left hip showing granulomatous dermatitis consistent with deep fungal infection.

A skin biopsy specimen obtained for histopathologic study and microbiological cultures revealed suppurative granulomatous inflammation in the deep dermis and hypodermis composed of lymphocytes, epithelioid macrophages, multinucleated giant cells and aggregations of neutrophils in the center of the granulomas (Fig. 2). Periodic acid-Schiff (PAS) stain showed round-shaped fungal structures (Fig. 3).

Fungal cultures were performed on Sabouraud chloramphenicol agar and Sabouraud chloramphenicol cycloheximide agar (Bio-Rad, Hercules, CA, U.S.A.), and incubated at 30 °C. After 10 days of incubation, velvety colonies developed on all media, which were olive-black with a blackish reverse. Microscopic examination of these colonies revealed brown septate hyphae, annellidic conidiogenous cells and oval conidia.

The isolate was subsequently referred to the Fungus Testing Laboratory in the Department of Pathology at the University of Texas Health Science Center at San Antonio (UTHSCSA DI 13-133), for antifungal susceptibility testing and identification by combined phenotypic characterization and DNA sequencing. Microscopic features were consistent with an *Exophiala* sp. and lack of growth at both 35 °C and 40 °C combined with a positive nitrate test suggested a species other than *E. dermatitidis*. For DNA sequencing, template DNA was prepared by subculturing the isolate onto PFA and incubating at 30 °C for 24 h. Hyphal elements were scraped from the agar surface, suspended in CPL-100 Buffer (VWR International INC, Radnor, PA), lysed in a Bead Beater instrument, and isolated manually by chloroform extraction method. Extracted DNA was used for PCR amplification of ITS and D1/D2 regions as described [30] with slight modification. PCR products were then sequenced using the ITS1 and ITS4 primers as well as NL1 and NL4 primers [22] at the UTHSCSA Molecular Diagnostics Laboratory. Sequences were assembled and analyzed using DNASTAR software (DNASTAR, Inc., Madison, WI) and queried in Genbank using the BLASTn algorithm at the NCBI site (www.ncbi.nlm.nih.gov). Sequences were also compared to those available in the CBS-KNAW Fungal Biodiversity Centre database (www.cbs.knaw.nl). The ITS sequence of this isolate showed 100% identity to both *Exophiala jeanselmei* (Genebank Accession no. AJ866273.1) and *E. oligosperma* (Genebank Accession no. AB480204.1), and the D1/D2 sequence showed 100% identity to *E. oligosperma* (CBS 265.49). By barcode analysis, there was 100% match with *E. oligosperma* (UTHSC 91-780) [31].

**Fig. 4:**
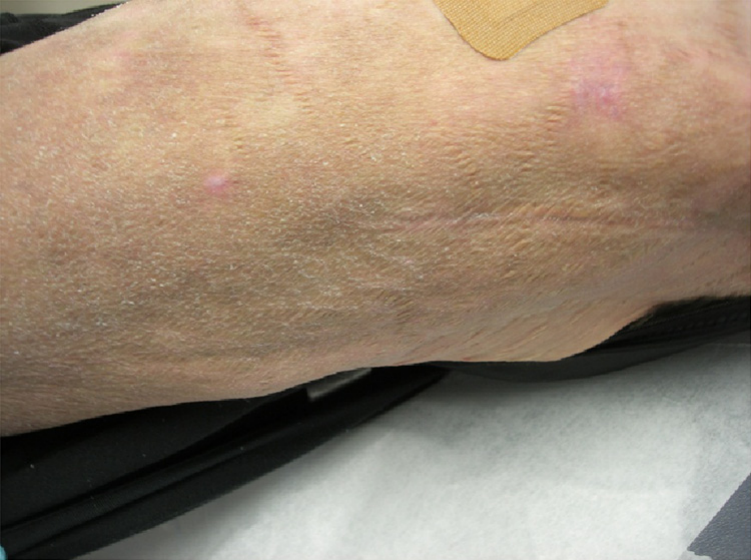
After voriconazole therapy – showing a decrease in size and resolution of her fungal infection.

Minimum inhibitory concentrations (MICs, in μg/ml) were performed via broth microdilution methods according to the CLSI M38-A2 guidelines, (CLSI M38-A2) and were reported as follows: amphotericin B 0.5 μg/ml, micafungin 0.25 μg/ml, posaconazole≤0.03 μg/ml, and voriconazole 0.125 μg/ml. The patient was started on oral voriconazole 200 mg twice daily. A follow-up assessment at 6 weeks showed interval reduction in the lesion size and tenderness (Fig. 4). At 12 weeks, the lesions had completely resolved and voriconazole was discontinued. She had no major side effects to the antifungal regimen. A 6-month follow up visit did not reveal any persistent lesions. Laboratory results, including complete blood count, chemistry and liver panel were normal throughout the treatment course.

## 3 Discussion

We searched the English-language literature published through May 2013 in the PubMed database. Relevant studies were identified using various key word combinations including “Exophiala, oligosperma, cutaneous” and “voriconazole.” No lower publication date limit was set. Six published cases of *E. oligosperma* human infections were ascertained (Table 1). Of these six cases, four showed susceptibility to voriconazole, with only one case describing successful clinical response to voriconazole, however, persistent lesions were present that required surgical excision [24] (Table 1).

*Exophiala* spp. are an emerging cause of skin and subcutaneous tissue infections, particularly in immunocompromised hosts. They have also been associated with systemic infections, including prosthetic valve endocarditis, dialysis-associated peritonitis, and disseminated infections [5–10]. These earlier reports originally attributed to *E. jeanselmei* most likely represent other species within the heterogeneous collection of yeast-like fungi [11]. Although the genus is often considered ubiquitous, several species occupy specific ecological niches contributing to their pathogenic potential. *Exophiala* spp. have been recovered from various climates, including hot, humid and oligotrophic environments, such as dishwashers, steam bath facilities, and bathrooms [12–14]. Other species associated with systemic infections include *E. dermatitidis*, *E. phaeomuriformis*, *E. lecanii-corni*, *E. bergeri*, *E. spinifera*, *E. mesophila*, and *E. attenuate* [2].

Subcutaneous phaeohyphomycosis usually presents as an asymptomatic, solitary nodule or abscess, usually found incidentally on an extremity. The infection commences as a small nodule that enlarges over months and may develop a pseudocapsule, giving a cyst-like appearance, termed “phaeohyphomycotic cyst” [18]. It has rarely been reported as multiple lesions [19]. In this context, it is noteworthy that in our case there was a cluster of inflammatory nodular lesions. The inoculation mechanism of the fungus in our index case is unknown; however, she gives a biologically plausible history of gardening with encounters of being pricked by a variety of plant thorns. In other cases, a history of prior trauma with splinters or other contaminated material occurred months before the appearance of the lesions [20]. Given the relatively nonspecific clinical features, subcutaneous phaeohyphomycosis may be confused with other entities and, thus, a high index of suspicion is required for early diagnosis.

The histopathologic picture is characterized by a granulomatous infiltrate, sometimes encapsulated by fibrous tissue [18,20]. Diagnosis depends on visualization of hyphal or yeast-like elements in tissue, and should be confirmed by isolation on culture and the morphological identification of the causal fungus. Moreover, differentiation down to the species level is recommended, as different species may have a different virulence or resistance to antifungal therapy [21]. *E. oligosperma* has an olive-black and velvety colony appearance, similar to that of other *Exophiala* spp. In our case, identification of the causative agent as *E. oligosperma* was achieved by a combination of phenotypic characteristics and DNA sequence analysis.

There are no set Clinical and Laboratory Standards Institute (CLSI) clinical breakpoints for antifungals against *Exophiala* species. However, extrapolating from MICs for other pathogens [3,4], we inferred activity for this isolate. As reported above, susceptibility testing by the CLSI M38-A2 guidelines demonstrated in vitro potency for various antifungals including amphotericin B and the azoles voriconazole and posaconazole. However, the treatment of choice for these rare infections has not yet been established in clinical studies.

The scarce data illustrate the importance of further investigation of more appropriate therapies. Amphotericin B, alone or in combination with flucytosine, or triazoles, such as voriconazole and itraconazole, have been used, but without completely satisfactory results [2]. Itraconazole has shown efficacy on some occasions, but its use is limited by its variable gastrointestinal absorption and alterations in cardiac conductivity [15]. Although tested only in vitro, posaconazole has also demonstrated good activity against clinical isolates of *E. oligosperma* [2,15]. In our patient, voriconazole was chosen due to her history of gastrointestinal intolerance to many medications and inability to reliably consume adequate dietary intake necessary for optimum absorption of posaconazole. Itraconazole was avoided due to her history of atrial fibrillation. A minimum of 3–4 months of therapy with voriconazole has been used [16], with up to 12 months in immunocompromised hosts [17]. Surgical excision is considered to be the treatment of choice when dealing with solitary, well-delimited lesions [18,23].

Unlike the response to voriconazole described by Kan et al. [24] the novelty in our index case is the complete resolution of symptoms without residual lesions. Furthermore, this previous case also involved the use of itraconazole and surgical intervention. Our case illustrates the efficacy of voriconazole as monotherapy without surgical intervention other than what was needed for her initial biopsy. That said, surgical intervention should be considered on a case-by-case basis. Although the case described by Kan et al. [24] involved combination antifungal therapies, clinical response was also seen at 3 months of therapy. In comparison to the other 3 case reports that showed susceptibility to voriconazole involving *E. oligosperma* cutaneous infections, none of those cases used voriconazole therapy.

In summary, we describe the first case of an *E. oligosperma* induced soft-tissue infection successfully treated with a 3-month course of voriconazole with complete resolution of all lesions noted with follow-up office visits. This may be likely secondary to the fact that our patient's skin lesions were probably more superficial and less extensive, when compared to the patients who failed different antifungal therapies with the same fungus. Healthcare providers should consider *E. oligosperma* when a cutaneous infection fails to respond to standard antimicrobials and broad, conventional antifungal therapy such as amphotericin B, fluconazole, or itraconazole. Although clinical experience with voriconazole in cases of disseminated phaeohyphomycosis caused by *E. oligosperma* is still scarce, voriconazole has shown the most promising results to date.

## Conflict of interest statement

There are none.

## Figures and Tables

**Table 1:** Cases of *Exophiala oligosperma* human infections with susceptibility to voriconazole (VCV)

**Cases**	**Year**	**Age**	**Gender**	**Predisposing factor**	**In-vitro susceptibility to VCV**	**Antifungal therapy used**	**Surgical intervention**	**MIC to VCV (μg/ml)**^a^	**Duration of therapy (months)**	**Response to surgical resection with or without therapy**
Bossler et al. [27]	2003	62	Male	Wegner's granulomatosis, olecranon bursitis	Yes	Amphotericin B	No	0.12	10	Not applicable
Al-Obaid et al. [28]	2006	3	Male	CAF^b^, leukemia	Yes	Amphotericin B itraconazole fluconazole	No	0.02	6	Not applicable
Gonzalez-Lopez et al. [26]	2007	72	Female	Renal transplant	Not performed	Itraconazole	No	Not performed	8	Not applicable
Badali et al. [29]	2011	20	Female	Chronic rhinosinusitis	Not performed	None	Yes	Not performed	0	Complete resolution
Tokuhisa et al. [25]	2011	57	Female	None	Not performed	Terbinafine	No	Not performed	6	Not applicable
Kan [3]	2013	71	Female	Wegner's granulomatosis	Yes	Voriconazole itraconazole	Yes	0.125	3	Complete resolution
Our index case	2013	50	Female	SLE^c^	Yes	Voriconazole	No	0.125	3	Not applicable

a μg/ml: micrograms per milliliter.

b CAF: catheter-associated fungemia.

c SLE: systemic lupus erythematosis.
